# Diverse Assemblage of Ediacaran fossils from Central Iran

**DOI:** 10.1038/s41598-018-23442-y

**Published:** 2018-03-22

**Authors:** Seyed Hamid Vaziri, Mahmoud Reza Majidifard, Marc Laflamme

**Affiliations:** 1 Department of Geology, North Tehran Branch, Islamic Azad University, P.O. Box 19585-851, Tehran, Iran; 2 Department of Chemical and Physical Sciences, University of Toronto Mississauga, 3359 Mississauga Rd, Mississauga, Ontario, L5L 1C6 Canada; 3 Research Institute for Earth Sciences, Geological Survey of Iran, P.O. Box, 13185-1494 Tehran, Iran

## Abstract

Reinvestigation of the Kushk and Chahmir areas (Bafq and Behabad regions) of central Iran has yielded a diverse assemblage of Ediacaran fossils, including several new species, just prior to the Cambrian explosion of complex animals. The Kushk series consists mainly of shallow marine carbonate deposits followed by deep-water calcareous marine shales. Ediacaran fossils occur commonly in the shale deposits and include biostratigraphically-important taxa *Cloudina* and *Corumbella*, which confirms a latest Ediacaran age for these deposits, the youngest examples of Kimberellomorphs (stem-group molluscs) that helps bridge the gap between their first occurrence in the middle-Ediacaran and the crown diversification in the Cambrian, and likely sponges, which are rare prior to the Cambrian.

## Introduction

The Ediacara biota represent an enigmatic group of large, multicellular, soft-bodied organisms with a global distribution in the latest Ediacaran (~570-541 Ma)^1^. Although their phylogenetic affinities are poorly resolved^2,3^, consensus is emerging that they most likely represent a diverse assemblage of stem and crown group animals in addition to extinct higher-order clades^4,5^. Their disappearance prior to the Cambrian explosion^4^ has been explained in many ways^6^, from an environmentally-driven terminal Ediacaran mass extinction^7,8^, to a preservational bias resulting from the fossilization (or lack thereof) of soft-tissues^9^. It has also been suggested that competition with emerging animals may have disrupted classic Ediacara biota, ultimately resulting in their extinction^10,11^. Importantly, the youngest Ediacaran assemblages (Nama Assemblage)^8,12–14^ are typically faunally depauperate^10^, dominated by a handful of modular Erniettomorpha^15^ and fractal Rangeomorpha^16^, and variety of tubular, often calcified, animals^17^.

**Figure 1:**
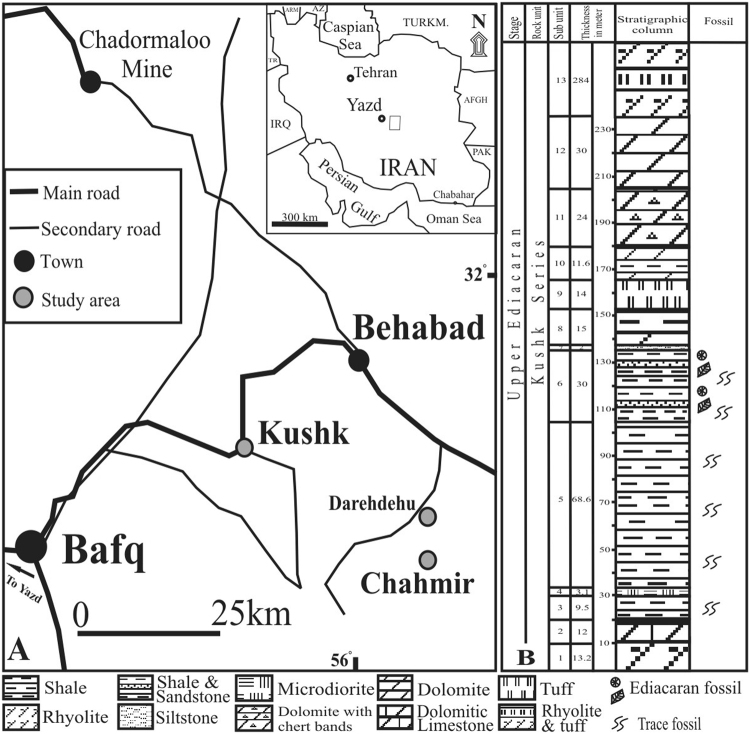
(**a**) Location of new field sites in central Iran. The Kushk Series accumulated in the Zarigan-Chahmir Basin (map by S.H.V., CorelDRAW Graphic Suite). (**b**) Stratigraphic column of the Kushk Series, which comprises 13 sub-units consisting of volcanic rhyolites, microdiorites, tuffs, dolomitic limestones, shales, and sandstones. Fossil occurrences are known from sub-units 6 (argillaceous shales).

Late Ediacaran fossils from the Lower Shale Member (Chopoqlu Shale) of the Soltanieh Formation of northern Iran^18^ and shale deposits of the Kushk Series in central Iran^19–21^ include the type material of *Permedusites changazensis* Hahn and Pflug 1980, however previous reports of *Dickinsonia?*, *Palaeoplatoda?*, *Pteridinium?*, *Spriggina?*, *Yazdia?*, and *Kushkia?* require further investigation in light of recent taxonomic and taphonomic studies^18–21^. Here we report the discovery of several new field sites from central Iran (Fig. 1a) that host a strikingly diverse assemblage of coexisting Ediacara biota and likely animals.

## Geological Setting

**Figure 2:**
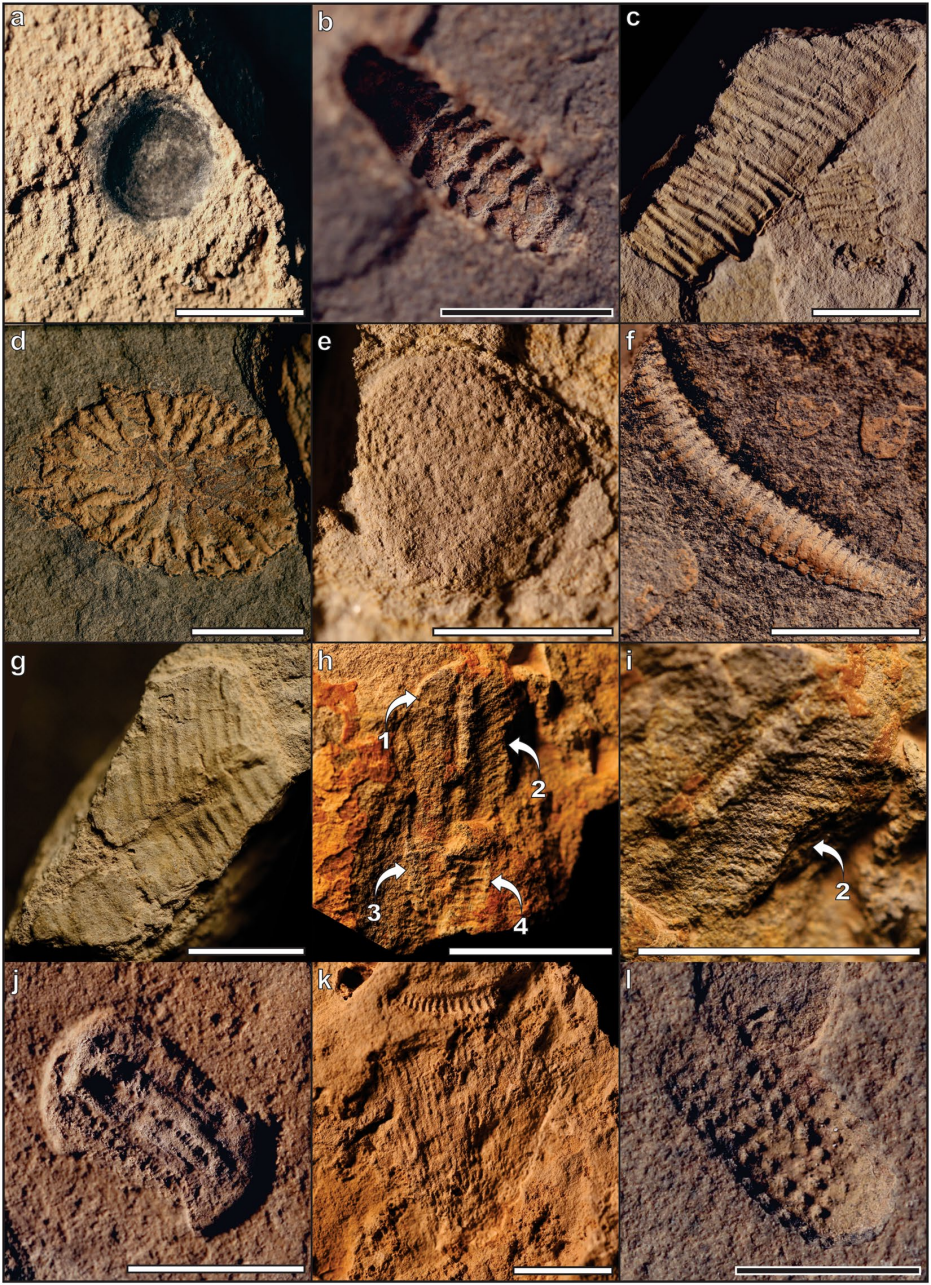
Ediacaran fossils from the Kushk Series, Kushk and Chahmir areas. (**a**) Organic-walled *Chuaria* (Ku/14/49), (**b**) *Cloudina* (deep negative impression, Ku/14/03a), (**c**) *Corumbella* (Ch/14/47), (**d**) *Persimedusites chahgazensis* (Ku/14/01), (**e**) *Kuckaraukia* (Ch/14/30), (**f**) unknown tubular organism (Ku/14/09a), (**g**) Erniettomorph (Ku/14/67), (**h**,**i**) Rangeomorph with at least 4 petaloid leaves (1-4) (Ku/14/15), (**j**) *Kimberella persii* n.sp. (Ch/14/62), (**k**) Possible trace fossil *Radulichnus* (Ku/14/114), (**j**) *Gibbavasis kushkii* n.sp. (Ch/14/56a). White (1 cm) and black (0.5 cm) scale bars.

The Kushk Series outcrops widely along the western margin of the Lut Block and the eastern margin of the Central Iranian Yazd Block. Sediments accumulated in the Zarigan-Chahmir Basin, which is bounded by the Kuhbanan Fault to the east and the Posht-e-Badam Fault to the west^22,23^. The Kushk Series reaches a maximum thickness of ~520 m in our sections, although the lower boundary is not exposed and the upper boundary is faulted with the Hashem Formation (Lower Cambrian). The Kushk Series comprises 13 sub-units consisting of volcanic rhyolites, microdiorites, tuffs, dolomitic limestones, dolomite, shales, and sandstones (Fig. 1b) interpreted as a deep, open-marine facies shallowing upwards into a carbonate platform^24^ resulting from a transgression-regression sequence. Of interest to this study is the fossiliferous sub-unit 6 (30–35 m), which is exposed in multiple areas (Kushk mine site, Chahgaz, Wedge, Chahmir, and Darehdehu). It is comprised of grey, thin-bedded argillaceous shales (containing trace fossils) with intercalation of grey, medium-bedded sandstones and massive sulfide mineralization containing pyritic intercalations, and green tuffs. These grey argillaceous shales can often weather to green (as seen in much of the study area). In the Wedge locality, sub-unit 6 is composed of grey argillaceous shales with intercalation of sandstones, tuff and dolomitic tuffs, while in the Chahmir area it consists of grey, thin-bedded siltstone and argillaceous shales with intercalation of tuff and very thin-bedded mudstone. This sub-unit can be correlated with the Lower Shale Member (Chopoqlu Shale) of the Soltanieh Formation^25^ in northern Iran. The extensive accumulations of volcanic material and tuffs, combined with the rapid perceived deepening of the platform, suggests that the Kushk basin may have represented an extensional rift basin related to opening of the Proto-Paleotethys sea^24^ in northeastern Gondwana. Geochronological constraints in the region are limited, with four ^207^Pb/^206^Pb dates of 595, 690 and 715 Ma (all ± 120 Ma)^26^ and 581 ± 8.6 Ma^27^ from the Kushk area, and a single ^207^Pb/^206^Pb date of 540.7 ± 4.8 Ma^27^ from the Chahmir area. Preliminary sampling of ash beds was unfortunately inconclusive; however, the discovery of classic Precambrian (*Chuaria* Fig. 2a) and terminal-Ediacaran index fossils (*Cloudina*, and *Corumbella*; Fig. 2b,c) support Hamdi and Jiang Zhiwen^28^ in assigning a latest Ediacaran (to earliest Cambrian) age. Of the three index fossils, *Chuaria* (n = 1) spans the greatest temporal interval (Tonian to Ediacaran) however it remains restricted to the Precambrian. The lightly-calcifying metazoan *Cloudina* (n = 37; Fig. 2b) is represented in these sections by external molds morphologically identical to *Cloudina* molds from Namibia^29^. Given the taxonomic difficulty of assigning species to dissolution impressions, the range of “cloudinids” is latest Ediacaran^30^ to possibly the basal Cambrian^31^. Finally, *Corumbella* (n = 108, Fig. 2c) is, to the best of our knowledge, restricted to the terminal Ediacaran^32,33^. As such, the only likely timeframe for the overlap of these three index fossils is latest Ediacaran.

## Results

Our new field sites represent the most diverse assemblage of late Ediacaran (Nama-Assemblage ~545-541 Ma) fossils known^6,10,12^, and includes the previously endemic Ediacaran fossil *Persimedusites chahgazensis* (n = 41, Fig. 2d;), which has been subsequently described (albeit identified as “Ediacaran discs”) from Argentina^34^. In addition to *Cloudina*, and *Corumbella*, Ediacaran fossils include the recently described *Kuckaraukia*^35^ (n = 4, Fig. 2e) from the Russian Urals, rare Erniettomorpha (n = 2, Fig. 2g) and Rangeomorpha (n = 1, Fig. 2h,i), and a diverse assemblage of tubular fossils (n = 16, Fig. 2f).

**Figure 3:**
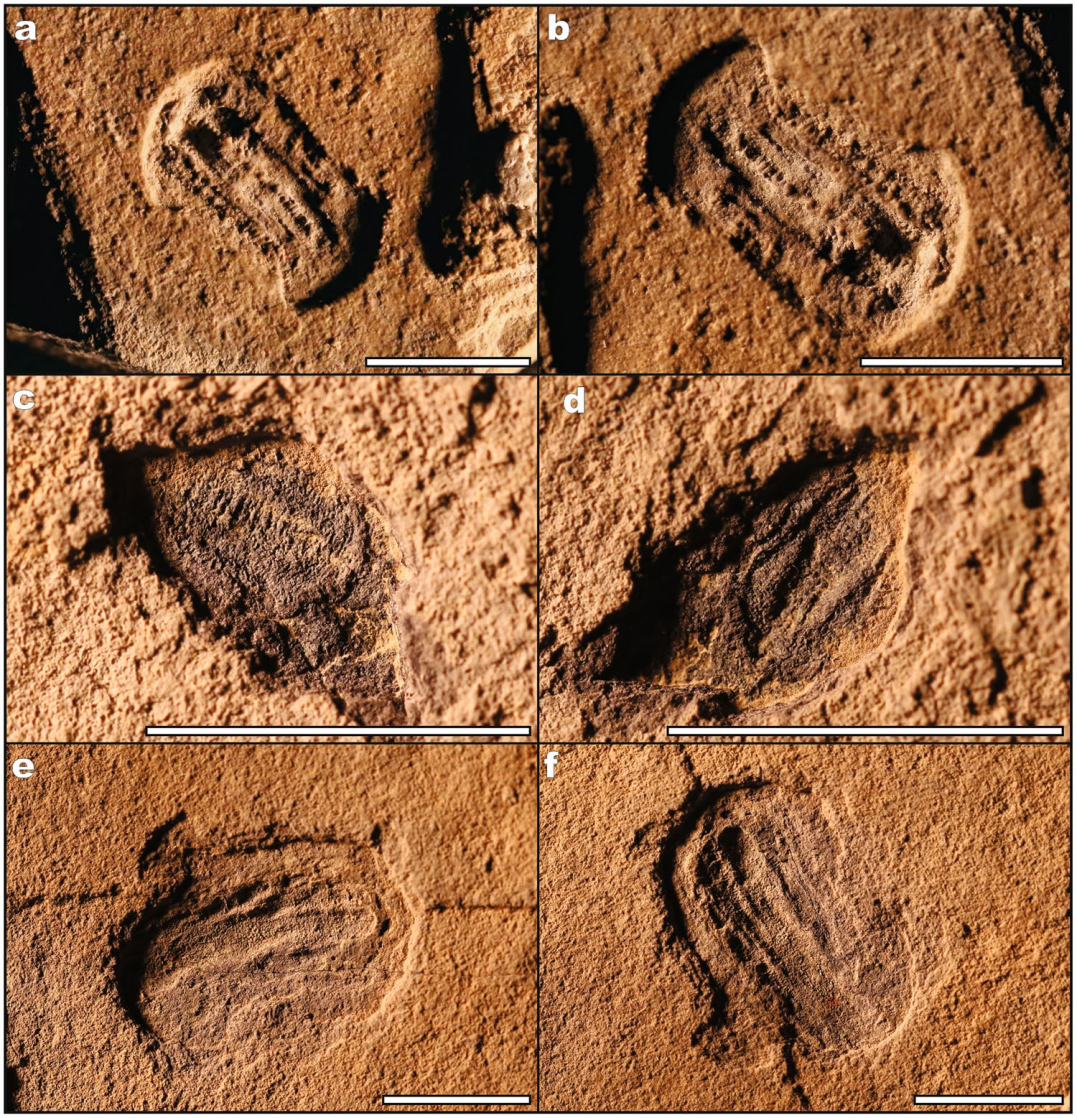
Specimens of *Kimberella persii* n.sp. from the Kushk Series, Chahmir area. (**a**,**b**) Specimen Ch/14/62, (**c**,**d**) specimen Ch/14/49a, (**e**,**f**) specimen Ch/14/49b. Scale bars: 1 cm.

**Figure 4:**
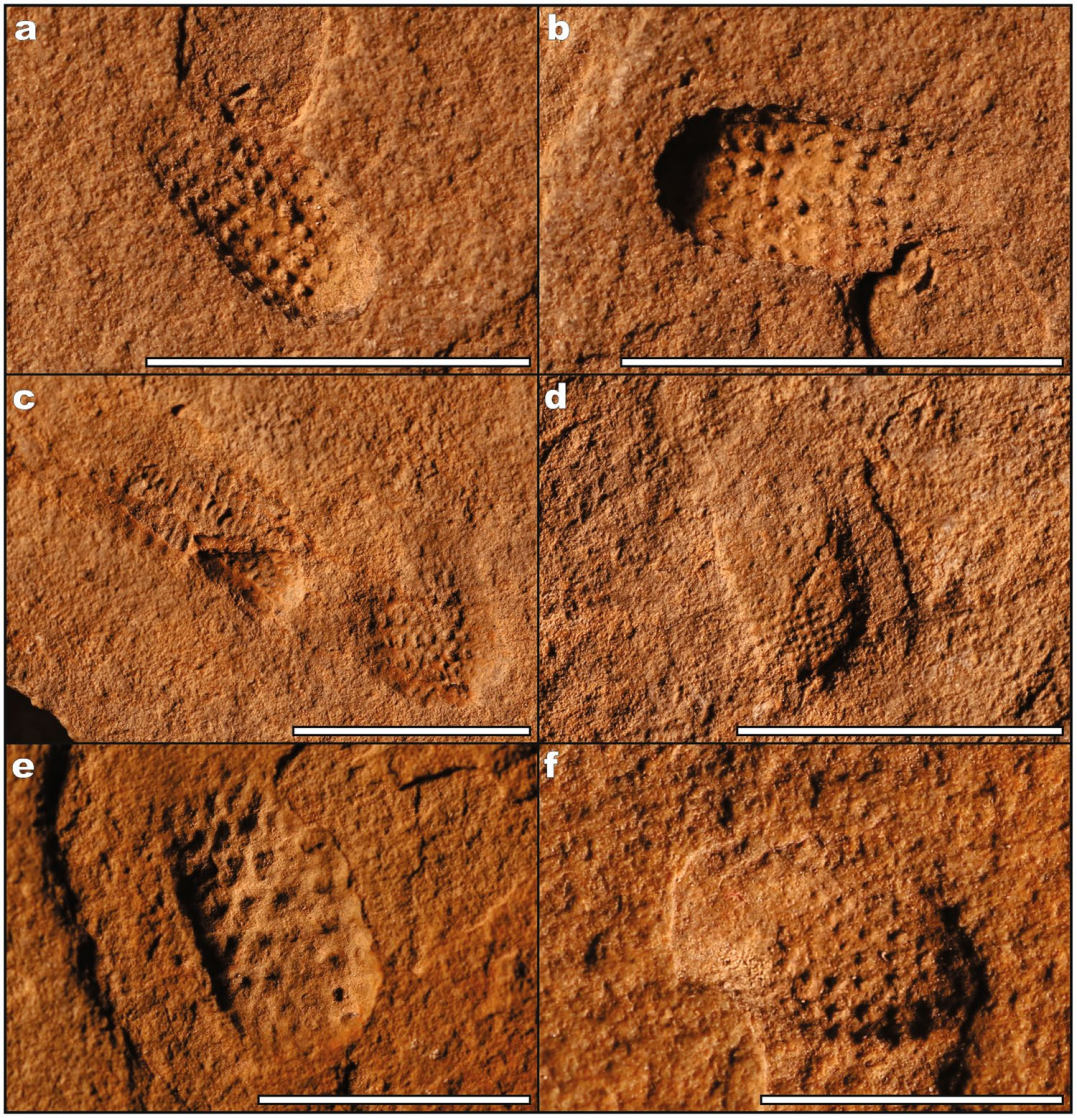
Specimens of *Gibbavasis* *kushkii* n.sp. from the Kushk Series, Chahmir area. (**a**,**b**) Specimen Ch/14/56a, (**c**,**d**) specimen Ch/14/56b, (**e**) specimen Ch/14/57a, (**f**) specimen Ch/14/57b. Scale bars: 1 cm.

Of particular importance are two new species that help bridge the gap between the high diversity Ediacaran assemblages of South Australia and Russia, with Cambrian assemblages of sponges and diverse crown bilaterians. *Kimberella persii* n.sp. (n = 9; Figs 2j, 3) consists of an oval to dumbbell-shaped fossil with anterior-posterior differentiation and clear segmentation along the midline. Although the impressions are compressed, at least three different sediment heights are found within the impression, implying differences in tissue lability and structural rigidity. *Kimberella persii* n.sp. shares the implied tissue differentiation and segmentation typical of *Kimberella quadrata*, however lacks the organic frill that outlines the periphery of the implied organic dorsal shell^36^ (Fig. 3). These characters suggest a bilaterian (stem mollusc) affinity^36^. *Gibbavasis kushkii* n.sp. (n = 9; Figs 2i, 4) consists of a small, goblet- to oval-shaped form preserved in negative epirelief with distinct rows of round protrusions, which originally represented external openings later infilled with sediment^37,38^. The overall columnar shape with distinct incurrent pores is suggestive of a poriferan-grade organism capable of effective filter feeding, however in the absence of siliceous or carbonaceous spicules, it is difficult to assign this species to an existing clade.

## Discussion

Recent studies of late-Ediacaran sections from Namibia^10,11^ and southwestern USA^8,14^ have demonstrated a distinctly depauperate global diversity of classic Ediacara biota (consisting almost exclusively of Erniettomorpha and Rangeomorpha), combined with an increase in diversity and abundance of mineralizing and organic tubular organisms^17^. The discovery of a high-diversity terminal Ediacaran population from central Iran is pivotal in assessing the nature of the end-Ediacaran extinction, in addition to a possible post White-Sea extinction of Ediacara biota^10,12^.

What is most striking about this end-Ediacaran assemblage is that it consists almost exclusively of animals. The rare and diminutive (in size) Ernitettomorpha (Fig. 2g) and Rangeomorpha (Fig. 2h,i) in this assemblage are dwarfed by the abundance of metazoans such as *Corumbella*^32^, *Cloudina*^39^, *Kimberella*, and *Gibbavasis*. The discovery of *Kimberella persii* extends the range of this group up to the Ediacaran-Cambrian boundary, filling the stratigraphic gap between the diverse White-Sea assemblage^6^ and the diverse crown molluscs known from many Cambrian Lagerstätte^40,41^. As such, the terminal Ediacaran of Iran showcases a changing Ediacaran ecosystem with a thriving metazoan community.

### Systematic Paleontology

Phylum **Mollusca** Linnaeus, 1758

Genus ***Kimberella*** Glaessner and Wade 1966

*Kimberella persii* n.sp.

(Figs 2j, 3)

#### Type species

*Kimberella quadata* Glaessner and Wade 1966.

#### Diagnosis

Elongate to oval-shaped, bilaterally-symmetrical form with segmented internal divisions represented as elliptical positive ridges along longest positive relief axis. Internal structure consists of four zones including central segments, internal, medial and marginal zones.

#### Description

Small (9–25 mm in length and 5–15 mm in width) oval (rarely cylindrical) fossil with anterior-posterior differentiation and prominent central segmented ridge (7–25 mm) consisting of 7–25 segments along the major axis. Adjacent to the internal segmented ridge are two parallel-sided smooth furrows 0.5–2 mm in width, surrounded by a slightly elevated and segmented 1–6 mm outer margin that strongly terminates at a distinct marginal rim.

#### Material

Nine specimens; figured specimens Ch/14/49a, b, 62 (Fig. 3), Chahmir area.

#### Locality

From gray argillaceous shales (sub-unit 6) of the Kushk Series in the Chahgaz (Dargazin) area near Kushk (Bafq) and Chahmir (Behabad), Central Iran.

#### Occurrence

*Kimberella* is known from the Ediacara Hills, Flinders Ranges (South Australia)^42^; Suz’ma, Karakhta and Solza rivers, Zimnii Bereg, White Sea (Russia)^36,43^; Kushk Series, Kushk area (Chahgaz (Dargazin) locale) in the Bafq region and Chahmir area in the Behabad region, Central Iran (this study).

#### Etymology

*Kimberella persii*, i.e. *Kimberella* from Persia.

Phylum **Uncertain**

Genus *Gibbavasis* n.gen.

*Gibbavasis* *kushkii* n.sp.

(Figs 2i, 4)

#### Diagnosis

Cylindrical to vase-shaped fossils with prominent positive nubs evenly spaced along parallel tracts around circumference.

#### Description

Small (4–14 mm long, 2–7 mm wide) vase-shaped, negative-relief impression consisting of regularly spaced positive relief and hemispherical bumps that run longitudinally as seven to nine rows across the width of the vase. Rows are also apparent latitudinally, resulting in a lattice-like appearance.

#### Remarks

Prominent positive-relief bosses likely represent a taphonomic inversion of an originally negative pit infilled with sediment. Pits may have served as an entryway for water currents. Specimens similar in construciton to the terminal Ediacaran fossil *Ausia* (from the Nama Assemblage), however *Gibbavasis* is noticeably smaller and vase-shaped rather than fan-shaped. The affinities of *Ausia* are unknown.

#### Material

Nine specimens; figured specimens Ch/14/56a, b, 57a, b (Fig. 4), Chahmir area.

#### Locality

From gray argillaceous shales (sub-unit 6) of the Kushk Series in the Kushk area (Chahgaz (Dargazin) locale in the Bafq region and Chahmir area in the Behabad region, Central Iran.

#### Etymology

*Gibbavasis* (Latin *Gibba* (bumpy) *vasis* (vase)) *kushkii* (from the Kushk Series of Central Iran).

## Methods

More than 250 Ediacaran specimens were collected (*in situ* and also from float) from the bed tops of the argillaceous shales (sub-unit 6; Fig. 1b) of the Kushk Series in Kushk area (Chahgaz (Dargazin) and Wedge locales in the Bafq region, and Chahmir and Darehdehu areas in the Behabad region, Central Iran. 95 specimens from the Kushk area and 150 specimens from the Chahmir area were investigated and identified. All specimens were washed and photographed, after which detailed morphological data were collected for all specimens.
